# Epidemiological and Radiomorphometric Aspects of the Accessory Mental Foramen in Brazilian Individuals: An Analysis by Cone Beam Computed Tomography

**DOI:** 10.4317/jced.61607

**Published:** 2024-11-01

**Authors:** Daniel Almeida Ferreira Barbosa, Eduardo Frederico Eduardo Maferano, Renata Cordeiro Teixeira, Lúcio Mitsuo Kurita, Alynne Vieira de Menezes Pimenta, Paulo Goberlânio de Barros Silva, Filipe Nobre Chaves, Marcela Lima Gurgel, Fábio Wildson Gurgel Costa

**Affiliations:** 1 DDS, MSc, PhD, Department of Oral and Maxillofacial Radiology, Federal University of Ceará, Ceará, Fortaleza, Brazil; 2 DDS, MSc, PhD student, Postgraduate Program in Dentistry, School of Dentistry, Federal University of Ceará, Ceará, Fortaleza, Brazil; 3 DDS, MSc, PhD, Department of Oral and Maxillofacial Radiology, School of Dentistry, Unifor University, Ceará, Fortaleza, Brazil; 4 DDS, MSc, PhD, Department of Oral Pathology, School of dentistry, Unichristus University Center, Ceará, Fortaleza, Brazil; 5 DDS, MSc, PhD, Department of Oral Diagnosis, Federal University of Ceará, Sobral Campus, Sobral, Ceará, Brazil; 6 Department of Dentistry, School of Health Sciences, Zambeze University, Tete, Mozambique

## Abstract

**Background:**

The accessory mental foramen is characterized by small foramina in the surrounding area of the mental foramen with communication with the mandibular canal. The aim of this research was to evaluate epidemiological and radiomorphometric aspects of the accessory mental foramen (AMF) using cone beam computed tomography (CBCT) in Brazilian individuals.

**Material and Methods:**

This was a cross-sectional, quantitative, retrospective descriptive study with 250 CBCT scans of dentate individuals in the anterior mandibular region, aged between 18 and 69 years. Presence/absence, linear measurements (length, height, and width), anatomical distances (to the vestibular, lingual, alveolar bone cortices, base of the mandible, and to the apex of the adjacent tooth) were evaluated concerning the accessory mental foramen.

**Results:**

Of the 250 scans, the mean age of the patients was 47.44±12.57 years, with 150 female and 100 male individuals. The total prevalence of AMF was 7.2% (n=18) of cases, with 5.2% (n=13) female individuals and 2% (n=5) male individuals. Bilateral presence of AMF was observed in one case (0.4%). Regarding laterality, AMF presence was observed in 10 cases (4.0%) on the right side and 8 (n=3.2%) on the left side. The mean distance from AMF to the mental foramen was 4.67 mm. The mean horizontal diameter was 1.06mm, and the mean vertical diameter was 0.95mm across the entire sample.

**Conclusions:**

The prevalence of the AMF was 7.2% with 95% confidence intervals (95% CI), showing a slight inclination towards female individuals.

** Key words:**Accessory mental foramen, mental foramen, cone beam computed tomography, anatomical variation.

## Introduction

The mental foramen (MF) is an important anatomical structure located in the anterolateral portion of the mandibular body, bilaterally between the roots of the first and second mandibular premolars or apical to the second premolar (1,2). Although the anatomy of the MF is widely recognized in humans and generally characterized as singular, anatomical variations may occur, including the presence of a double or multiple mental foramina, which is designated as an accessory mental foramen. The accessory mental foramen is characterized by small foramina in the surrounding area of the MF with communication with the mandibular canal, resulting from the branching of the mental nerve before exiting the mental foramen. This anatomical variation is rare, with a prevalence estimated between 1.4% and 13.7% (3-6).

Differences in the prevalence of the accessory mental foramen may be attributed to ethnic factors and the techniques used for its identification. Studies have shown that the accessory mental foramen is less frequent in populations of white Americans and Asian Indians than in African Americans and Nazca Indians, being more common in men. Additionally, it is noted that when accessory mental foramina are present, they tend to be predominantly unilateral, in the right hemimandible, and generally located posterior and inferior to the mental foramen (3,7-9).

Various methods have been used to study the accessory mental foramen (AMF), from gross anatomical dissection to radiological techniques and observation of dry mandibles. However, anatomical methods face limitations due to the small sample size. Panoramic radiographs may present difficulties in visualizing the AMF due to its size and geometric distortion of the images. In this context, cone-beam computed tomography (CBCT) has been recommended as a tool with significant accuracy for evaluating the AMF non-invasively, providing precise anatomical details as it is a three-dimensional examination with superior linear measurement accuracy compared to panoramic radiography (4,9-12).

The exact understanding of the location of the accessory mental foramen is particularly relevant in dental practice, especially in local anesthesia and surgical procedures in this region, such as genioplasty, removal of block bone grafts, and especially, dental implant placement (1,11,13). Therefore, the aim of this research was to evaluate epidemiological and radiomorphometric aspects of the accessory mental foramen (AMF) through cone-beam computed tomography (CBCT) in Brazilian individuals.

## Material and Methods

-Study Design and Ethical Aspects

This is a cross-sectional study with CBCT scans obtained from routine procedures at different dental imaging centers. This research was conducted following the recommendations of the Strengthening the Reporting of Observational Studies in Epidemiology (STROBE) for observational studies (14).

-Sample

Considering a prevalence of 100% for the mental foramen (15) and a three-year collection period, it was estimated necessary within a population of 1380 tomographies to evaluate 173 tomographies to obtain a sample that represents, with a precision of 5% and a confidence of 95%, the images of the service n=Z^2 .P.(1−P).N α^2 /Z^2 .P.(1−P)+(N−1).e^2. An additional 10% was added to this, totaling a minimum of 191 tomographies. From a database with 300 tomographies, a simple randomization mechanism was drawn for image selection. If any image did not meet the eligibility criteria, the immediately subsequent registration image would be selected.

-Eligibility Criteria

CBCT scans of individuals of both sexes, aged between 18 and 69 years, involving the anterior region of the mandible, and presenting all teeth in the interforaminal region were included. On the other hand, tomographic exams that evidenced: suggestive images of pathological alterations (e.g., cysts and tumors), fractures, and syndromes that altered the local bone architecture; severe bone loss that made the adequate study of any of the anatomical repairs evaluated in this study impossible; image artifacts affecting diagnostic quality, such as dental implants and plates and screws for fracture fixation were excluded.

-Image Acquisition and Evaluation

CBCT images obtained from two imaging centers that followed the eligibility criteria adopted in this investigation were evaluated, being coded by numbers to maintain anonymity. The tomographic exams were acquired through the I-Cat and I-Cat Next Generation devices (Imaging International Sciences, Hatfield, Pennsylvania, USA), and the patients remained in a static position (standing or sitting) following the manufacturers’ recommendations for these tomographs and with appropriate protocols for clinical indications (Supplementary Material 1). The tomographies were analyzed by a single observer, an expert in Dental Radiology, previously trained for data collection. The evaluations were performed on the same computer (Apple Inc., MacBook Pro model, Intel Core i7 processor, 2.3 GHz) using Imaging Studio software version 3.4 (Anne Solutions, Brazil) (16) under low-light conditions.

-Data Collection and Analysis

The three-dimensional volumes were traversed through reconstructions in the three different planes, aiming to identify any accessory foramina. The accessory mental foramina (AMF) were classified regarding absence/presence, diameter, and distance from the AMF to the MF. The AMF was classified regarding the number, as 0 – absent; 1 - present; and when present, the number of AMF found from 1 to 4, according to the study by do Carmo Oliveira *et al*. (17). After identifying the accessory foramen, the shortest distance between the accessory mental foramen and the mental foramen was measured using a methodology developed by the author.

**Figure 1:**
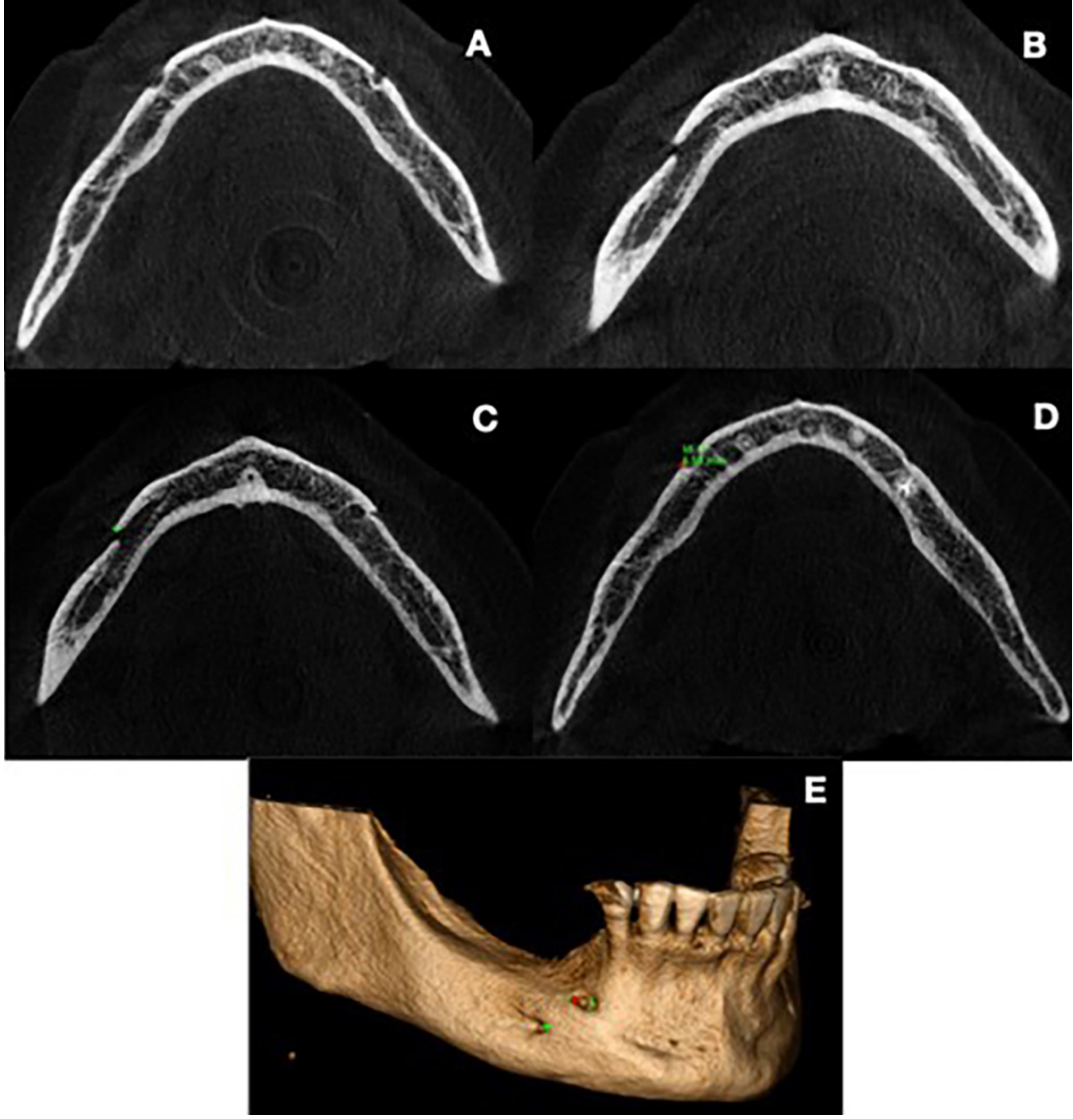
A - Presence of the mental foramen (MF). B - Presence of the accessory mental foramen (AMF) posterior to the MF. C - Marking of the most anterior point of the AMF. D - Marking of the most posterior point of the MF. E - 3D reconstruction showing the marked points, in green for the AMF, and in red for the MF.

Once the AMF was visualized (Fig. 1A), the shortest distance between the AMF and the MF was measured (Fig. 1B). For this purpose, the most anterior point of the AMF closest to the MF (Fig. 1C) and the most posterior point of the MF closest to the AMF (Fig. 1D) were marked. With this, the measurement was visualized in the 3D reconstruction (Fig. 1E).

The position of the AMF relative to the MF was classified into four possible directions: 1) distal-inferiorly (DI); 2) distal-superiorly (DS); 3) medio-inferiorly (MI); 4) medio-superiorly (MS). Furthermore, laterality regarding the presence of additional foramina was recorded, being classified as unilateral or bilateral.

-Study Calibration and Error

The examiner underwent intra-examiner calibration testing prior to data collection, evaluating 30 images with a 15-day interval in which tomographic exams were randomly selected. To evaluate reproducibility errors, the following analyses were performed: 1. Intraclass Correlation Coefficient (ICC) to assess systematic errors related to numerical data, considering values up to 0.5 as poor discordance, between 0.5 and 0.75 moderate, between 0.75 and 0.9 good, and above 0.9 is considered excellent Goyushov *et al*. (18); 2. Dahlberg’s formula to assess casual errors of the measurements made. Regarding ICC, the ICC model of two-way random effects with a 95% confidence interval was used, considering values with *p*<0.05 as satisfactory. To evaluate possible technical method errors, Dahlberg’s formula (19) was used.

-Statistical Analysis

Data were tabulated in Microsoft Excel and exported to the Statistical Package for the Social Sciences (SPSS) version 20.0 for Windows adopting a confidence of 95%. The characteristics of the AMF were expressed in the form of absolute and percentage frequency and analyzed by Pearson’s chi-square test. Quantitative measurements were expressed as mean and standard deviation, subjected to Kolmogorov-Smirnov normality test, and analyzed by Mann-Whitney, Wilcoxon, and Spearman correlation tests (non-parametric data).

## Results

A total of 250 images were evaluated, with 150 from female subjects and 100 from male subjects. The mean age of the patients was 47.44±12.57 years, with no difference between sexes (*p*=0.434), and the majority of patients were under 50 years old.

The overall prevalence of the AMF was 7.2% (n=18) of cases, with 13 (5.2%) individuals being females and 5 (2%) males with 95% confidence intervals (95%CI). Regarding  Table 1, we observed the occurrence of up to 1 AMF per side in the evaluated tomographic exams. Bilateral presence of the AMF was observed in one case, and there were no cases of two AMFs on the same side. Regarding laterality, the presence of AMF was observed in 10 cases (4.0%) on the right side and 8 (3.2%) on the left side. The location of the AMF was mainly observed on the MS aspect, followed by DI, MI, and DS. On the right side, the primary locations of AMF were in the MI, MS, and DI aspects (30% each), followed by DS (10%). The highest relative frequencies on the left side were in the MS location (37.5%), followed by DS and DI (25% each), and MI (12.5%). There was no statistically significant difference regarding prevalence variables (*p*=0.518) or location (*p*=0.785) of AMF ( Table 1).

With regard to linear measures ( Table 2), the MF - AMF distance, with a mean of 4.67 mm, received special consideration. The distance measured in males was 4.90 ± 2.82 mm, whereas in females it was 4.61 ± 3.16 mm. The average vertical diameter was 0.95 mm, with females exhibiting greater values (0.95 ± 0.43 mm) than males (0.94 ± 0.36 mm). In the same way, females had a larger horizontal diameter (1.07 ± 0.46 mm) than men (1.05 ± 0.41 mm).

## Discussion

The prevalence of AMF varies widely depending on the population studied, ranging from 1.4% to 13.7% (3,6). In this study, a prevalence of 7.2% was found, corroborating with other studies conducted in the Brazilian population (20,21), contrasting with studies that observed 3.0% and 3.2% (11,22). These findings suggest that geographical location within the same country may influence the prevalence of AMF.

In the present study, the accessory mental foramen was defined as a buccal foramen with continuity to the mandibular canal, and the nutrient foramen was distinguished as a buccal foramen without such continuity, according to Naitoh *et al*. (13). Studies conducted in Peru, Turkey, and China (6,23,24) found AMF prevalence between 10.50% and 13.7%. Thus, when compared to other worldwide populations, Brazilians apparently have a lower prevalence of AMF.

Some studies suggest that gender may influence AMF prevalence as demonstrated by Han *et al*. (8) and Li *et al*. (9) who found a statistically significant difference between genders, being more frequent in men. In the present study, there was a higher prevalence of AMF in women compared to men without statistically significant differences, which is conflicting with the study by Aytugar *et al*. (6) who reported a higher occurrence in men, also without statistically significant differences.

AMF may be present on one side only or on both sides; in this study, a vast majority of cases were unilateral with only one bilateral case observed, representing one case (6.25%) in 16 individuals, being 0.4% of the total sample. A similar finding was observed by Lam *et al*. (25) in which AMF was observed in 0.4% of individuals. Some studies did not show the occurrence of bilateral AMF (1,22,23).

The accessory mental foramen (AMF) communicates with the mandibular canal (MC), allowing the emergence of accessory vessels and nerves that vascularize and innervate the adjacent teeth. The identification of the AMF is of utmost importance in clinical practice, especially in the presence of multiple accessory foramina, as their location may impact the insertion of dental implants and other surgical procedures in the anterolateral region of the mandible. According to Naitoh *et al*. (13), it is possible to find up to two AMFs on the same side.

Another important aspect regarding AMF besides the number is its location, as depending on its position, there is a greater or lesser risk of injury in invasive procedures. The classification of AMF position proposed by Zmyslowska-Polakowska *et al*. (1) described two possible situations, anterior and posterior, in which they reported 54.6% and 28.6% of cases located anteriorly to the MF and 45.5% and 71.4% posteriorly to the MF, respectively.

Currently, most studies classify the position of AMF in the anteroposterior direction, as well as inferosuperior, which was adopted in this study. According to Kalender *et al*. (26), the most prevalent position was anteroinferior (33%), followed by inferior, superior, and posterosuperior (16.6%), and Aytugar *et al*. (6) reported the posterinferior position with the highest prevalence in 35% of cases.

This study showed AMF present mesiosuperiorly to the MF in 33.3% of cases, corroborating with the findings of Imada *et al*. (11) also in a Brazilian population who reported two cases (n=50%) just above the MF and the other two (n=50%) anterior to the MF.

Anterior locations of AMF are clinically important because the region between the MFs is a commonly used area for dental implant placement and is considered an excellent bone graft donor site (27). Injuries to AMF can cause trans- and post-operative bleeding, as well as neurosensory disturbances (27).

Aytugar *et al*. (6) showed in their study a distance from AMF to MF of 3.22mm in men and 3.05mm in women. This study found on the right side an average of 4.68mm and on the left side of 4.53mm with no statistically significant difference between sides, as well as no difference between genders. Wei *et al*. (24) and Li *et al*. (9) reported in their studies the distance from AMF to MF of 5.1 ±1.4 mm and 5.32±1.55mm, respectively, in Chinese populations, Zmyslowska-Polakowska *et al*. (1) observed in Polish individuals this distance being 2.86 ±1.34mm. Kalender *et al*. (26) evaluating a Turkish population found an AMF to mental foramen distance greater than the others cited earlier being 6.6±4.2mm. These findings suggest that ethnicity may have some influence on the AMF to mental foramen distance.

The diameters of AMF were smaller than previous studies, with mean vertical diameter of 0.95 mm. Aytugar *et al*. (6) and Li *et al*. (9) reported this measurement with an average of 1.36±0.54 mm and 1.23±0.37mm respectively. Regarding the horizontal diameter, it was smaller (1.05±0.41 mm) in males when compared to females (1.07±0.46 mm) with no statistically significant difference. Li *et al*. (9) showed in their study an average horizontal diameter of 1.38±0.47 mm. Therefore, it has been demonstrated that AMF presents smaller dimensions when compared to MF in several studies.

This study has some limitations. The sample used is a subpopulation of northeastern Brazil, which prevents its representativeness for the Brazilian population as a whole. Additionally, no sexual dimorphism was observed in the anatomical repairs evaluated. Therefore, further studies are suggested in different populations for a more comprehensive analysis of the anatomical repair addressed in this work.

## Conclusions

In the analyzed sample, the prevalence of Accessory Mental Foramen (AMF) was 7.2% with 95% confidence intervals (95%CI), showing a slight inclination towards female individuals. Most cases were unilateral, mainly located on the right side, especially in patients under 50 years old. No significant differences were observed between the right and left sides regarding the mean distance from AMF to MF, as well as the horizontal and vertical diameters. These results provide a contribution to the understanding of AMF anatomy, being useful for healthcare professionals. However, further research is needed for a more complete understanding of this structure.

## Figures and Tables

**Table 1:** Sample characteristics and prevalence of the AMF regarding the right and left sides.

	Side		
	Right	Left	p-Value
AMF - Prevalence			
0	240 (96.0%)	242 (96.8%)	0.518^a^
1	10 (4.0%)	8 (3.2%)	
2	0 (0.0%)	0 (0.0%)	
AMF - Location			
DS	1 (10.0%)	2 (25.0%)	0.785^a^
MI	3 (30.0%)	1 (12.5%)	
MS	3 (30.0%)	3 (37.5%)	
DI	3 (30.0%)	2 (25.0%)	

*p<0.05, a Pearson’s chi-square test (n, %). Legend: 0: absent; 1: present; 2: presence of two accessory mental foramina on the same side; AMF: accessory mental foramen; DS: distal-superiorly; MI: medio-inferiorly; MS: medio-superiorly; DI: distal-inferiorly.

**Table 2:** Mean ± SD of linear measurements in millimeters related to the AMF.

	Mean measurement			Coefficient of variation		
	Total	Female	Male	p-Value^a^	Female	Male	p-Value^b^
Mean							
MF-AMF distance	4.67±3.00	4.61±3.16	4.90±2.82	0.827	35.9%	33.2%	0.179
Vertical diameter	0.95±0.40	0.95±0.43	0.94±0.36	0.615	45.5%	38.1%	0.163
Horizontal diameter	1.06±0.43	1.05±0.41	1.07±0.46	0.708	39.3%	42.8%	0.158

*p<0.05, a Mann-Whitney test; b Levene’s test (mean ± SD) Legend: MF: mental foramen; AMF: accessory mental foramen; SD, standard deviation.
